# Single-Cell RNA Sequencing-Based Computational Analysis to Describe Disease Heterogeneity

**DOI:** 10.3389/fgene.2019.00629

**Published:** 2019-07-12

**Authors:** Tao Zeng, Hao Dai

**Affiliations:** Key Laboratory of Systems Biology, Institute of Biochemistry and Cell Biology, Chinese Academy of Sciences, Shanghai, China

**Keywords:** cellular heterogeneity, complex diseases, single-cell RNA sequencing, network, integration

## Abstract

The trillions of cells in the human body can be viewed as elementary but essential biological units that achieve different body states, but the low resolution of previous cell isolation and measurement approaches limits our understanding of the cell-specific molecular profiles. The recent establishment and rapid growth of single-cell sequencing technology has facilitated the identification of molecular profiles of heterogeneous cells, especially on the transcription level of single cells [single-cell RNA sequencing (scRNA-seq)]. As a novel method, the robustness of scRNA-seq under changing conditions will determine its practical potential in major research programs and clinical applications. In this review, we first briefly presented the scRNA-seq-related methods from the point of view of experiments and computation. Then, we compared several state-of-the-art scRNA-seq analysis frameworks mainly by analyzing their performance robustness on independent scRNA-seq datasets for the same complex disease. Finally, we elaborated on our hypothesis on consensus scRNA-seq analysis and summarized the potential indicative and predictive roles of individual cells in understanding disease heterogeneity by single-cell technologies.

## Introduction

It is known that an adult human body consists of trillion cells of different types and origins, and each of them plays its respective role in the body system. These cells can be viewed as basic but essential biological units supporting different body states, e.g., health, disease, or the response to therapy. Decades ago, the low resolution of cell isolation and measurement technologies limited our understanding of the cell-specific molecular profiles and their importance in cellular systems, causing humans to always underestimate disease heterogeneity.

In recent years, the establishment and the rapid growth of single-cell sequencing technology have led to the efficient and inexpensive identification of molecular profiles of individual cells (Bose et al., 2015; Baran-Gale et al., 2018; Svensson et al., 2018). In particular, the transcription of single cells (Wu et al., 2014; Ziegenhain et al., 2017) is a novel and fast evolving field. Single-cell RNA sequencing (scRNA-seq) attracts increasing attention to the identification and characterization of cells on an individual level rather than on a population level (Saliba et al., 2014; McDavid et al., 2016; Raj et al., 2018; Torre et al., 2018).

The research field of single cells, e.g., identifying cell types, recognizing cell markers, and tracing cell origins, is currently undergoing rapid development. New knowledge on cells can improve our understanding of biological systems by changing our perspective from the traditional population level to the individual cellular level. It can further provide novel insights into old biological and biomedical questions (Raj et al., 2018). For example, with scRNA-seq data rather than bulk transcriptome data, we can detect genes with conserved expression levels across individual cells (Lin Y. et al., 2017). Single-cell transcriptomics could even uncover the diverse transcriptional states of immune cells and their coordination during immune responses (Vegh and Haniffa, 2018). In addition, simultaneous measurements of transcription along with genomic and epigenetic profiling at the single-cell level (Clark et al., 2016) is expected to be developed soon and will provide groundbreaking biological insights into these basic blocks building the biological body (Hemberg 2018).

In this quickly evolving field, many reviews have focused on the biotechnological applications of scRNA-seq and in silico gene expression analysis. The program goals of the Common Fund-supported Single Cell Analysis Program from the National Institutes of Health point out the impact of resolving tissue heterogeneity at the cellular level (Roy et al., 2018). Different scRNA-seq protocols have their strengths and disadvantages under respective settings (Saliba et al., 2014; Bacher and Kendziorski, 2016). The pre-processing approaches of sparse and row-rank scRNA-seq data (Zhang L. et al., 2018), normalization methods (Vallejos et al., 2017), and batch effect corrections (Dal Molin and Di Camillo, 2018; Haghverdi et al., 2018) have all been carried out for a wide range of comparisons and evaluations. Finally, the cell type clustering algorithms, cell marker identification, and cell trajectory reference also have their target-specific evaluation approaches for the deconvolution of biological system heterogeneity (Menon 2018; Papalexi and Satija, 2018). In addition, integrative impacts of whole scRNA-seq protocols and analysis methodologies have undergone in-depth assessments (Dal Molin et al., 2017; Svensson et al., 2017; Todorov and Saeys, 2018).

These current developments and achievements of scRNA-seq motivated us to investigate the individual cell types, cell signatures, cell origins in time and space, and cell communication strategies. Meanwhile, as a novel method, its robustness under different conditions (e.g., when applied to different datasets) will determine its actual practical potential in major research programs (e.g., the Precision Medicine Initiative or the Human Brain Project) (Poo et al., 2016; Sankar and Parker, 2016) or in clinical applications (e.g., diagnosis or prognosis of complex diseases) (Zeng et al., 2016). Thus, in this review paper, we discussed scRNA-seq from the point of view of experiments and computation. Then, on independent scRNA-seq datasets for the same complex disease (i.e., diabetes), we compared several state-of-the-art scRNA-seq analysis frameworks mainly by the robustness of their performances in the identification of cell types and markers. Lastly, we elaborated on our hypothesis on consensus scRNA-seq analysis and summarized the potential indicative and predictive roles of characteristic cells in understanding disease heterogeneity by single-cell technologies.

## Materials and Methods

A recent review has demonstrated the principle and potential of scRNA-seq in a wide range of studies, including development, physiology, and disease (Potter 2018). It concluded that the data noise and cell number are the main limitations in scRNA-seq studies, and many research fields would benefit from its continuous development. In contrast, this work concentrated on the scRNA-seq-based study from the two angles of experiments and computation. Especially, the robustness of scRNA-seq under changing conditions will decide its practical potential, e.g., in precision medicine. Thus, different from a previous report (Potter 2018), we further compared several state-of-the-art scRNA-seq analysis frameworks and included our hypothesis on the performance consensus.

### scRNA-seq-Associated Biological Experiments

scRNA-seq is becoming a widely used genome-wide technology to detect cellular identities and dynamics, e.g., cell subpopulations, cell state marker genes and pathways, cell state transitions, and cell trajectories (Nguyen et al., 2018). This sustained improvement of the sensitivity, flexibility, and efficiency of scRNA-seq will help to resolve many biological and biomedical research questions on the individual cell level.

On the one hand, the rapid development of experimental protocols of scRNA-seq expands the measurement of mRNA levels to many associated fields of study (Fuzik et al., 2016; Hashimshony et al., 2016; Ilicic et al., 2016; Bagnoli et al., 2018; Han et al., 2018; Hayashi et al., 2018; Sasagawa et al., 2018). Especially, scRNA-seq applications have provided new insights into conventional biological questions, e.g., cellular heterogeneity. New cell types have been more widely recognized than previously expected (Burns et al., 2015; Usoskin et al., 2015; Rheaume et al., 2018), and gene expression levels corresponding to old and new cell types have uncovered many biological functions and mechanisms that were overlooked in conventional cell population studies (Nelson et al., 2016; Li H. et al., 2017); single-cell transcriptomic characteristics can reveal more time-dependent features of a biological system (Zeisel et al., 2015; Zeng et al., 2017; Lescroart et al., 2018; Liu D. et al., 2018), whereas the pseudo-time of single cells would mimic the actual dynamic biological process (Kowalczyk et al., 2015; Cacchiarelli et al., 2018). Taking all of the above novelties together, we can deepen our understanding on the complex mechanisms underlying cell-to-cell variation. These complex dynamic responses are controlled by regulatory cell-to-cell communication, which is also responsible for cellular heterogeneity (Shalek et al., 2014).

#### Measuring Regulatory Elements in a Single Cell

Cell-specific transcriptional signals might be regulated by the high-order structural folding of nucleosomes (Nagano et al., 2017; Lando et al., 2018), which can be investigated by combining scRNA-seq with other single-cell approaches (Stevens et al., 2017; Liu T. et al., 2018; Mezger et al., 2018). Of note, current scRNA profiling methods usually destroy cells during the analysis process, hindering the measurement of temporal gene expression changes. However, some information on biological dynamics will always be present in the data. For example, the continuum of molecular states in a population can reflect the trajectory or pseudo-time of a typical cell, so various methods increase their power by reconstructing the trajectory by quantification of a group of cells in multiple static snapshots (Weinreb et al., 2018).

#### Measuring Post-transcriptional Regulations in a Single Cell

Understanding nongenetic cellular heterogeneity will help to characterize complete biological mechanisms in live cells, but little knowledge is available on the heterogeneity of regulatory modifications between individual cells. For example, microRNAs (miRNAs) are small RNAs that regulate gene expression in a post-transcriptional manner and might reduce cell-to-cell variability on the protein level by repressing mRNA translation or promoting mRNA degradation. Although the wet experimental evidence for the roles of miRNA in individual cells is limited, great efforts have been made to investigate such regulatory modifications in single cells (Fan et al., 2015). For instance, single-cell Quartz-Seq technology was developed to identify different kinds of nongenetic cellular heterogeneity in a quantitative manner (Sasagawa et al., 2013). Single-cell small RNA sequencing and analysis techniques have supplied much evidence that miRNAs could be potential molecular biomarkers for indicating the type and state of particular cells (Faridani et al., 2016). Moreover, using a combination of scRNA-seq data and mathematical modeling, it is also possible to detect key miRNAs as cell type-specific post-transcriptional regulators (Rzepiela et al., 2018).

#### Measuring Upstream Regulatory Factors in a Single Cell

Individual cells within different subpopulations can show significant variations when responding to external stresses, but the nature of this cellular heterogeneity is not clear, especially the remarkable alterations in the transcriptional architecture (Xue et al., 2013; Edsgard et al., 2016; Gasch et al., 2017). Fortunately, scRNA-seq provides high resolution to genetics by linking phenotypes to cell-specific gene functions, and the genetic screening of single cells can even be realized now (Birnbaum 2018; Raj et al., 2018). For example, the Perturb-seq was designed to combine scRNA-seq and CRISPR-based perturbations to detect individual perturbations causing target gene changes, gene signature appearances, genetic interaction rewiring, and cell state transitions (Dixit et al., 2016), e.g., discovering previously unknown immune circuits (Jaitin et al., 2016). Next, the allele-sensitive scRNA-seq could recognize clonal and dynamic monoallelic expression patterns (Reinius et al., 2016) or analyze allele-specific cis-control in genome-wide expressions (Deng et al., 2014; Jiang et al., 2017). Besides, focusing on the quantitative trait locus (QTL), the computational tool demuxlet was implemented to perform expression QTL (eQTL) analysis, which can identify natural genetic variation within multiplexed droplet scRNA-seq to evaluate cell type-specific gene expression changes (Kang et al., 2018). Similarly, some new cell type-specific “co-expression QTLs” have even been detected according to the genetic variants, significantly altering co-expression relationships (van der Wijst et al., 2018).

#### Measuring Downstream Regulation in a Single Cell

The cell-to-cell regulatory communication plays important roles in cellular diversity across diverse biological systems, which is an important factor in the evolution of observed cell types. scRNA-seq provides a powerful tool to analyze particular regulatory mechanisms and their downstream influence in a corresponding subset of cells (Chu et al., 2016; Korthauer et al., 2016; Enge et al., 2017; Severo et al., 2018). For example, the integration of transcription factor expression, chromatin profiling, and sequence motif analysis can be effective to identify the cell-specific genomic regulation underlying cell-specific gene expression (Sebe-Pedros et al., 2018). Similarly, the integration of information about single-cell transcriptomics and cell-free plasma RNA provides the potential to uncover longitudinal cellular dynamics of cells in complex biological processes or pathological development (Tsang et al., 2017). Next, a two-part method combining a generalized linear model and gene set enrichment analysis on single-cell data provided evolutionary insights in gene co-expression by experimental treatments (Finak et al., 2015). In addition, benefitting from time-course data obtained by scRNA-seq, it is possible to characterize the fate decision and transcriptional control of self-renewal, differentiation, and maturation of particular cells (Su et al., 2017), and transient cellular states corresponding to asynchronous cellular responses can be observed under conditional perturbations (Rizvi et al., 2017).

### scRNA-seq-Associated Analytic Computations

As seen in the above summary, scRNA-seq technologies are swiftly developing. They are greatly beneficial to the investigation of transcriptional landscapes at the single-cell level, where they are able to profile cell-to-cell variability in cell populations and characterize unexpected heterogeneity of transcription in originally thought homogeneous cell populations. Although many computational methods for analyzing scRNA-seq data have been extensively developed, tested, and validated on simulated datasets, scRNA-seq protocols are still complex so that bias will easily occur in downstream analysis. In fact, computational models and tools available for the design and analysis of scRNA-seq experiments (**Table 1**) have their advantages and disadvantages in various settings, and many questions have yet to be solved in this exciting area (Bacher and Kendziorski, 2016). Similar to other high-throughput sequencing technologies, the general actions on scRNA-seq data include several key steps before the follow-up analysis for single cells (Jia et al., 2017; Li Y. H. et al., 2017; McCarthy et al., 2017; Chen W. et al., 2018; Vu et al., 2018), i.e., pre-procession (e.g., zero imputation) (Li and Li, 2018; Van den Berge et al., 2018), quality control (e.g., variation analysis) (Brennecke et al., 2013; Ding et al., 2015; Jiang et al., 2016; Eling et al., 2018; Lu et al., 2018), normalization (Bacher et al., 2017; Cole et al., 2017; Haghverdi et al., 2018; Tian et al., 2018), and visualization/simulation (Zappia et al., 2017). Although scRNA-seq studies have provided revolutionary tools to assist researchers to address scientific questions previously hard to investigate directly, several computational challenges are beginning to arise.

**Table 1:** List of computational tools for single-cell RNA sequencing (scRNA-seq) analysis.

Category	ID	Access	Code and citation
Pre-procession	scater	Bioconductor	R (McCarthy et al., 2017)
	scPipe	Bioconductor	R (Tian et al., 2018)
	GRM	http://wanglab.ucsd.edu/star/GRM	R (Ding et al., 2015)
Cell clustering	SAFEclustering	http://yunliweb.its.unc.edu/safe/	R (Yang et al., 2018)
	DendroSplit	Github	Python (Zhang J. et al., 2018)
	clusterExperiment	Bioconductor	R (Risso et al., 2018)
	scmap	Bioconductor	R (Kiselev et al., 2018)
	scVDMC	Github	Matlab (Zhang H. et al., 2018)
	CIDR	Github	R (Lin P. et al., 2017)
	scClustBench	http://www.maths.usyd.edu.au/u/SMS/bioinformatics/software.html	R (Kim et al., 2018)
	SNN-Cliq	http://bioinfo.uncc.edu/SNNCliq	Matlab & Python (Xu and Su, 2015)
Cell marking	MAST	Github	R (Finak et al., 2015)
	SC2P	Github	R (Wu et al., 2018)
	DEsingle	Bioconductor	R (Miao et al., 2018)
	powsimR	Github	R (Vieth et al., 2017)
	BPSC	Github	R (Vu et al., 2016)
	Sincell	Bioconductor	R (Julia et al., 2015)
Cell ordering	dynverse	Github	R (Saelens et al., 2018)
	Progra	Github	R (Gong et al., 2018)
	p-Creode	Github	Python (Herring et al., 2018)
Pipeline	SINCERA	https://research.cchmc.org/pbge/sincera.html	R (Guo et al., 2015)
	SCell	Github	Exe (Diaz et al., 2016)
	Falco	Github	Python (Yang et al., 2017)
	ASAP	Github	R & python (Gardeux et al., 2017)
	SIMLR	Github	R & Matlab (Wang et al., 2017; Wang B. et al., 2018)
	SEURAT	http://satijalab.org/seurat/	R (Butler et al., 2018)
	Monocle	Bioconductor	R (Trapnell et al., 2014; Qiu et al., 2017a; Qiu et al., 2017b)
	DPT	http://www.helmholtz-muenchen.de/icb/dpt	R & Matlab (Haghverdi et al., 2016)
B-cell receptor reconstruction	VDJPuzzle	bitbucket	R & Python (Rizzetto et al., 2018)
	bracer	Github	Python (Lindeman et al., 2018)
Network inference	SCODE	Github	R (Matsumoto et al., 2017)
	LEAP	CRAN	R (Specht and Li, 2017)

#### Challenge of Cluster Analysis of Single Cells

The detection of cell types from heterogeneous cells is an important step in the development of scRNA-seq data analysis in biological research (Marinov et al., 2014; Lin C. et al., 2017; Jin et al., 2018; Kiselev et al., 2019). Different methods use distinct characteristics of data and gain varying outcomes in terms of both the number of clusters and the cluster assignment of cells (Ntranos et al., 2016; Kim et al., 2018; Risso et al., 2018). Many approaches, such as SAFE clustering (Yang et al., 2018), DendroSplit (Zhang J. et al., 2018), scmap (Kiselev et al., 2018), MetaNeighbor (Crow et al., 2018), scVDMC (Zhang H. et al., 2018), CIDR (Lin P. et al., 2017), SC3 (Kiselev et al., 2017), scLVM (Buettner et al., 2015), and RaceID (Grun et al., 2015), have been developed to promote the efficiency of clustering single cells. They promote the clustering consensus, interpretability, subjectivity, comparability, and replicability. However, the biological significance, number estimation, and computational speed of such clustering analysis still require significant improvements (Duan et al., 2018).

#### Challenge of Identity Analysis of Single Cells

scRNA-seq has brought transcriptome research to a higher resolution as the “up or down” expression pattern can be examined at the single-cell level (Chen L. et al., 2018; Xie et al., 2019). The projection of high-dimensional data into a low-dimensional subspace will be a powerful strategy for mining such extensive data (Zeng et al., 2016; Yip et al., 2018; Yu and Zeng, 2018). Statistic-based approaches, such as PowsimR (Vieth et al., 2017), BPSC (Vu et al., 2016), Linnorm (Yip et al., 2017), and Oscope (Leng et al., 2015), have been established to evaluate differential expression among individual cells. Especially, latent factor-based analysis will be useful to find hidden biological signals and corresponding gene components from scRNA-seq samples (Buettner et al., 2017; Yu, 2018). However, to guarantee the biological meaning of detected cell identities, it is still necessary to discriminate the real and dropout zeros in scRNA-seq data (Miao et al., 2018). It is also essential to identify the combination of binary and continuous regulation in individual cells (Wu et al., 2018) and to integrate the nonlinear projection with prior-known biological knowledge (Li X. et al., 2017).

#### Challenge of Trajectory Analysis of Single Cells

The single-cell experiments provide a great chance to rebuild a sequence of changes in a dynamical process of the biological system from individual “snapshots” of cells (Matsumoto et al., 2017; Gong et al., 2018). The construction of a pseudo-temporal path as cell orders would be a useful way to characterize dynamical gene expression in a heterogeneous cell population, assuming the existence hypothesis of gradual transition of the cell transcriptome (Specht and Li, 2017; Herring et al., 2018; Shindo et al., 2018; Strauss et al., 2018). For example, based on the minimum spanning tree approach, the Tools for Single Cell Analysis is developed for in silico pseudo-time reconstruction in scRNA-seq analysis (Ji and Ji, 2016). As an iterative supervised learning algorithm, FateID can recognize the cell fate preference by quantifying the lineage-specific probabilistic biases (Herman et al., 2018). By unsupervisedly selecting feature genes and judging the location and number of branches and loops, SLICER is able to infer highly nonlinear trajectories (Welch et al., 2016). However, many opportunities still exist to develop these current methods, particularly detecting complex trajectory topologies, linking pseudo-time and real-world time, determining baseline points, estimating transition possibility, and recognizing progression trends with tipping point (Zeng et al., 2013).

#### Challenge of Origin Analysis of Single Cells

The origin and nature of signals leading to pattern formation and self-organization is an essential question in developmental or stem cell biology. The answer would be recovered from the gene expressions of individual cells with spatial locations in a particular tissue (Vergara et al., 2017; Chen Q. et al., 2018). On the one hand, from the technological point of view, several methods have been designed for recording the spatial information of cells. The spatial transcriptomic technology and computational deconvolution can be combined to detect distinct expression profiles corresponding to different tissue components (Berglund et al., 2018). One technique that performs RT-LAMP reactions on a histological tissue section can preserve the original spatial location of the nucleic acid molecules to become an effective tissue analysis tool (Ganguli et al., 2018). Another technique is based on a panel of zonated landmark genes, where the lobule coordinates of mouse liver cells can be inferred according to their transcriptome, whereas the zonation profiles of all liver genes can also be characterized with high spatial resolution (Halpern et al., 2017). On the other hand, from the analytic point of view, supervised methods have been shown to be efficient, inferring the potential spatial distribution of cells. On the foundation of a reference gene expression database, e.g., the gene expression atlas for positional gene expression profiles within cells, an scRNA-seq-based high-throughput method has been applied to identify the spatial origin of cells (Achim et al., 2015). Obviously, spatial labeling technologies still need further technological developments for more easy and accurate testing, and the spatial classification and prediction of cells require more elaborate and efficient mathematical and computational models.

#### Challenge of Integrative Analysis of Single Cells

Understanding the genetic and cellular processes and programs driving the differentiation of diverse cell types and organ formation is a major challenge in developmental biology (Kelsey et al., 2017, Velten et al., 2017, Duren et al., 2018, Liu L. et al., 2018). Frameworks and software are required to perform dimension reduction, clustering, and visualization on scRNA-seq data to improve biological interpretability (Gardeux et al., 2017; Wang et al., 2017). Numerous methods have been implemented for analyzing scRNA-seq data in a whole life-cycle manner (Guo et al., 2015; Diaz et al., 2016; Leng et al., 2016; Yang et al., 2017). SparseDC solves a unified optimization problem so that it can carry out three tasks simultaneously, e.g., identifying cell types, tracing expression changes across conditions, and identifying marker genes for these changes (Barron et al., 2018). BigSCale implements a scalable analytical framework to handle millions of cells, so it can overcome large data challenges by the directed down-sampling strategy on index cell transcriptomes (Iacono et al., 2018). In addition to these usual analytic routines for conventional targets, more diverse integration models are required for data-driven, model-driven, hypothesis-driven, and combinatory bioinformatics mining in single-cell data.

### Understanding Disease Heterogeneity by scRNA-seq Analysis

For questions in the biological and biomedical fields, human cancers are especially considered complex ecosystems where the basic elements (cells) exist in different disease states characterized by phenotypes and genotypes. As is well known, conventional methods have their limits when measuring and quantifying the diverse tumor (cell) composition in patients, e.g., traditional bulk expression profiles have to average the cells within each tumor. Nowadays, scRNA-seq provides a powerful technique to detect critical cell differences and deconvolve such cellular heterogeneity in disease tissues. Therefore, one important benefit obtained from scRNA-seq is the possibility to decipher tumor architecture (Cloney 2017), so that it might overcome intratumoral heterogeneity, which hampers the success of precision medicine and is therefore a huge challenge in cancer treatment (Patel et al., 2014; Kim et al., 2016; Zong, 2017). Actually, in the context of cancer, mRNA can be used to identify malignant cells and diverse tumor-tissue compositions; such tumor compositions could indicate the cancer-associated cells and types determining tumor characteristics (Young et al., 2018). Thus, scRNA-seq-based methods could be widely applied in clinical decision support (Tirosh et al., 2016a; Filbin et al., 2018; Krieg et al., 2018; Pellegrino et al., 2018).

- *Tumor mechanism investigation*. One general framework can be used to decipher differences between multiple classes of human tumors by decoupling cancer cell genotypes, phenotypes, and the composition of tumor microenvironment (Venteicher et al., 2017). One single-cell analysis method has provided some insights into the cellular architecture of oligodendrogliomas and their function in development regulation, which potentially is compatible with the cancer stem cell model and its consideration in disease management (Tirosh et al., 2016b).
- *Tumor subtype recognition*. To deconvolve the cellular composition of a solid tumor from bulk gene expression data using reference gene expression profiles from tumor-derived scRNA-seq data, many cell types or subtypes must be identified accurately (Schelker et al., 2017). For example, one scRNA-seq study of triple-negative breast cancer identified the individual subpopulations with respective gene expression phenotypes and corresponding genotype driver candidates, whose associated signature genes can predict long-term outcomes (Karaayvaz et al., 2018).
- *Tumor immune therapy*. Single-cell analyses have suggested distinct patterns in the tumor microenvironment, e.g., the breast cancer transcriptome has shown a wide range of intratumoral heterogeneity that is reshaped by both immune and tumor cells in a closely communicated microenvironment at a single-cell resolution (Chung et al., 2017). An unbiased scRNA-seq analysis has detected human dendritic cells and several monocyte subtypes in the human blood to permit more accurate immune monitoring in health and disease (Villani et al., 2017). In a more special field, the single-cell transcriptional information in B-cell lineages might have broad applications involved in vaccine design, antibody development, and cancer treatment (Rizzetto et al., 2018; Upadhyay et al., 2018).
- *Tumor virus-environment recognition*. Indeed, the interaction between a host and a pathogen is a highly dynamical process, so the potential association between a pathogen and cancer is worthy of profound investigation. An scRNA-seq-based method, scDual-Seq, has been proposed to capture host and pathogen transcriptomes simultaneously (Avital et al., 2017). In different mouse models, the hypothetical virus-host interaction events have been found to play some key regulatory role in virus phenotypes involved in complex diseases by tracking viral RNA at single-cell resolution within the immune system (Douam et al., 2017).

Of course, the translational usage of scRNA-seq is not limited to the field of tumor biology or complex human diseases; it is expected to have great potential and to enjoy a wide range of applications in biological and biomedical fields, such as infant development, health and wellness, and disease monitoring.

### Design of Hypothesis and Theory Study on scRNA-seq Analysis Robustness

As is well known, scRNA-seq analysis is used to compare the expression levels of multiple genes at single-cell resolution (Tang et al., 2009). Different from the conventional population-based biological technologies for gene expression measurement (e.g., bulk gene expression), scRNA-seq is able to distinguish the expression differences between individual cells rather than tissues. With the continuous development of such technology, the testing cost is decreasing, whereas the number of cells that can simultaneously be tested is increasing exponentially. Some recent reviews have summarized these technological developments and protocol improvements in scRNA-seq analyses (Svensson et al., 2017; Ziegenhain et al., 2017; Svensson et al., 2018). An inspiring observation is that the number of tested cells and the number of detected genes can vary significantly depending on the corresponding experimental platforms. For example, SMART-seq2 is able to detect about 10,000 genes and achieve the highest accuracy, but the number of cells analyzed by this method is only 100 to 1,000 (Picelli et al., 2013; Picelli et al., 2014). In contrast, Drop-seq is able to test more than 10,000 cells simultaneously, but the number of genes detected is usually less than 5,000 (Macosko et al., 2015). Recently, several commercial platforms, such as 10X Genomics Chromium, Fluidigm C1, and Wafergen ICELL8, were available for scRNA-seq analysis with the capability to measure hundreds to millions of cells through a simple and fast workflow.

Researchers are usually required to select the suitable experimental protocol to design the follow-up scRNA-seq analysis based on corresponding biological questions:

- If one aims to discover new cell types with distinct expression patterns, more cells should be tested because it is impossible to find rare cell types from only a few hundred cells by chance.
- If one aims to analyze the changes in gene expression between different cell types or developmental stages or to analyze the gene interactions to find some key regulatory genes, more genes have to be measured with high accuracy.
- If one aims to analyze particular cell types by isolating a subset of cells for sequencing, fluorescence-activated cell sorting or a similar technology needs to be used to select the cells with cell type-specific cell surface markers.

To evaluate and investigate the robustness of different scRNA-seq analysis methods, we have carried out two comparisons on multiple scRNA-seq datasets.

The aim of the first comparison is to discuss the experimental factors for scRNA-seq analysis. As is well known, the accuracy of RNA-seq data analysis is dependent on the experimental methods, especially the sequencing depth and dropout rate. To test these experimental factors before further evaluation, we compared four datasets on two different experimental platforms: GSE81608 (Xin et al., 2016) and GSE83139 (Wang et al., 2016) on an Illumina HiSeq 2500 and GSE86469 (Lawlor et al., 2017) and GSE81547 (Enge et al., 2017) on an Illumina NextSeq 500. All of these datasets come from the single-cell studies of human pancreatic islet cells so that their computational results will be comparable, and the number of clusters for each method was fixed to be the same as the number of biological classes corresponding to each dataset, as shown in **Table 2**.

**Table 2:** Clustering performances of four datasets with different experiment methods represented as adjusted rand index (ARI).

	GSE81547	GSE83139	GSE81608	GSE86469
Experiment platforms	NextSeq 500	HiSeq 2500	HiSeq 2500	NextSeq 500
Number of cells	2,282	635	1,600	617
Number of detected genes per cell on average	3,281	5,638	5,706	8,339
Number of potential cell types*	6	8	4	7
Hierarchical clustering	0.34	0.25	0.46	0.63
k-means	0.34	0.27	0.44	0.48
tSNE+k-means	0.37	0.34	0.54	0.72
SIMLR	0.34	0.32	0.51	0.61
SNN-Cliq	0.10	0.31	0.05	0.61
SEURAT	0.31	0.31	0.45	0.89

*GSE81547 includes alpha cells, beta cells, delta cells, acinar cells, mesenchyme cells, and ductal cells. GSE83139 includes alpha cells, beta cells, delta cells, PP cells, acinar cells, mesenchyme cells, ductal cells, and dropped cells. GSE81608 includes alpha cells, beta cells, delta cells, and PP cells. GSE86469 includes alpha cells, beta cells, delta cells, PP cells, acinar cells, stellate cells, and ductal cells.

The aim of the second comparison is to discuss the analytic approaches for scRNA-seq analysis. The performance of dissimilar methods on different real datasets of the same complex disease is important to evaluate, because performance robustness will be strictly required for biomedical studies and applications. Thus, we have employed several widely used methods in a few public scRNA-seq datasets from complex disease studies, which are listed in **Table 3**. According to the above summary, we actually evaluated the performances on cell cluster, cell identity, and cell trajectory. These methods’ parameter settings are listed in the supplementary files (**Supp 1**).

- For cell clustering analysis, traditional methods, such as hierarchical clustering, k-means, and scRNA-seq-induced SIMLR (Wang et al., 2017; Wang B. et al., 2018), SNN-Cliq (Xu and Su, 2015), and SEURAT (Butler et al., 2018) have been evaluated and compared.
- For cell pseudo-time analysis, the Monocle (Trapnell et al., 2014; Qiu et al., 2017a; Qiu et al., 2017b) and diffusion pseudo-time (DPT) (Haghverdi et al., 2016) have been tested and compared.

**Table 3:** Summary of evaluation datasets on human complex diseases.

Data ID	Purpose	Platform	#scRNA-Seq	#Class
GSE69405	scRNA-seq identifies subclonal heterogeneity in anticancer drug responses of lung adenocarcinoma cells	HiSeq 2500	176	3
GSE73121	scRNA-seq in optimizing a combinatorial therapeutic strategy in metastatic renal cell carcinoma	HiSeq 2500	118	3
GSE81608	scRNA-seq on human islet cells revealing type 2 diabetes genes	HiSeq 2500	1600	4
GSE83139	scRNA-seq of the human endocrine pancreas	HiSeq 2500	635	8

Of note, to quantitatively measure and compare the analysis accuracy of cell clusters from different methods, the conventional adjusted rand index (ARI) is applied. Given a dataset of *n* cells, the experimentally determined cell types are *X* *_1_*, *X* *_2_*, …, *X* *_r_* and the calculated clusters are *Y* *_1_*, *Y* *_2_*, …, *Y* *_s_*. The number of cells that belong to cell type *X* *_i_* is denoted as *a* *_i_*, the number of cells that belong to cluster *Y* *_j_* is denoted as *b* *_j_*, and the number of cells that belong to both *X* *_i_* and *Y* *_j_* is denoted as *n* *_ij_*, which means *n* *_ij_* = |*X* *_i_* ∩ *Y* *_j_*|. Then, the ARI is calculated as follows:

$$\mathrm{ARI}=\frac{\Sigma_{ij}\left({}_{2}^{n_{ij}}\right)-\left[\Sigma_{i}\left({}_{2}^{a_{i}}\right)\cdot \Sigma_{j}\left({}_{2}^{b_{j}}\right)\right]/\left({}_{2}^{n}\right)}{\frac{1}{2}\left[\Sigma_{i}\left({}_{2}^{a_{i}}\right)+\Sigma_{j}\left({}_{2}^{b_{j}}\right)\right]-\left[\Sigma_{i}\left({}_{2}^{a_{i}}\right)\cdot \Sigma_{j}\left({}_{2}^{b_{j}}\right)\right]/\left({}_{2}^{n}\right)}.$$

## Results and Discussion

### Experimental Factors for scRNA-seq Analysis

The experimental processes of the four datasets presented in **Table 2** are briefly summarized below.

1. For GSE81608 (Xin et al., 2016), islets were handpicked and enzymatically digested; during RNA *in situ* hybridization, the cells were permeabilized and hybridized with combinations of mRNA probes and a multiplex fluorescent kit was used to amplify the mRNA signal. Sequencing was performed on an Illumina HiSeq2500 in rapid mode by multiplexed single-read run with 50 cycles.
2. For GSE83139 (Wang et al., 2016), human islets require careful sample acquisition and preparation; the SMART-seq method was used for first-strand cDNA synthesis and polymerase chain reaction (PCR) amplification. All of the libraries were sequenced on the Illumina HiSeq 2500 with 100 bp single-end reads.
3. For GSE86469 (Lawlor et al., 2017), islets are systematically acquired, processed, and dissociated; then, single-cell processing is carried out on the C1 single-cell Autoprep system. All of the sequencing was performed on an Illumina NextSeq500 using the 75-cycle high-output chip.
4. For GSE81547 (Enge et al., 2017), the experimental models and human pancreas or islet samples were conducted in accordance with guidelines; during flow cytometry, isolated human islets were dissociated into single cells by enzymatic digestion using Accumax (Invitrogen). Next, single-cell RNA-seq libraries were generated as described in the literature, and barcoded libraries were pooled and subjected to 75 bp paired-end sequencing on the Illumina NextSeq instrument.

Of course, the whole experimental process should be consistent; however, the scRNA-seq wet experiments in different studies were conducted with different parameters and under different circumstances, which are worthy of future evaluation. Although sequencing platforms are only one part of the scRNA-seq experiment, we tried to include them for the comparison study in this work. In **Table 2**, we see that there is no obvious performance difference between two experiment platforms; however, the accuracy (i.e., ARI) seems to increase when the number of detected genes becomes large for almost all of the tested methods, which is consistent with a previous conclusion (Potter, 2018) and implies that the influence of sequencing depth is very important in the experimental protocol for follow-up data analysis. Of note, the parameter setting for each compared method in this work is outlined in the supplementary files (**Supp 1**).

### Analytic Approaches for scRNA-seq Analysis

**Figure 1:**
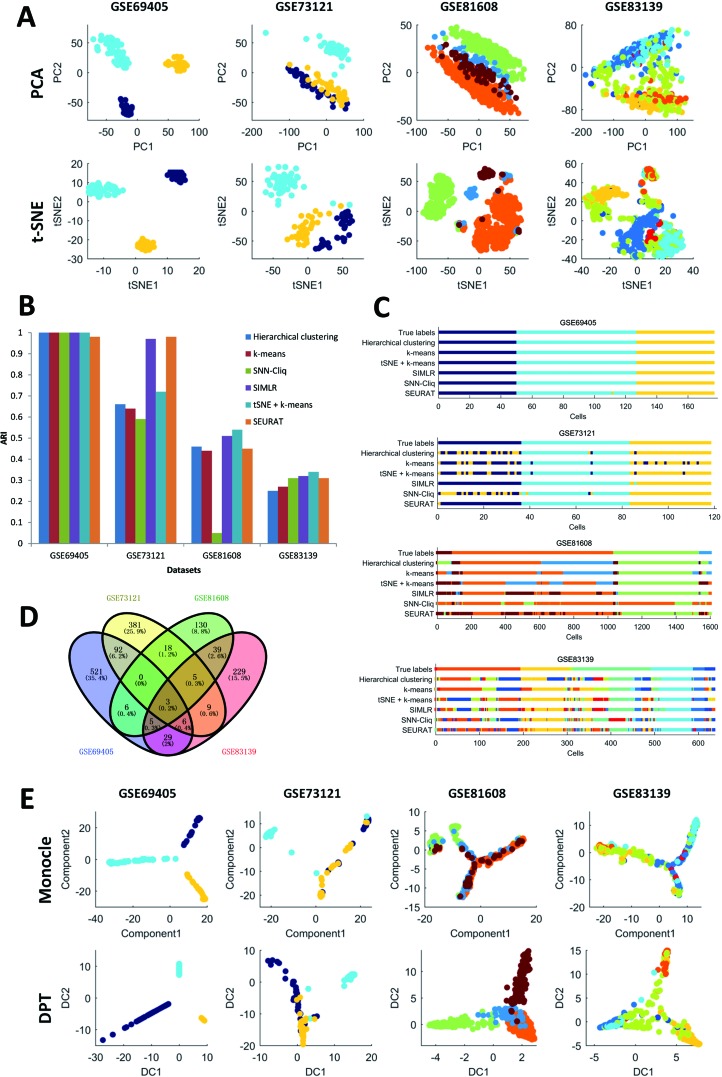
Summary of performance comparison.

First, it can be seen that the datasets after dimension reduction by t-distributed stochastic neighbor embedding (tSNE) (Maaten and Hintton, 2008) exhibit better performances in conventional k-means clustering than the initial dataset, which is due to the noise reduction of scRNA-seq data. Dimension reduction can be used in the visualization of such phenomena, which reduces one dataset from high-dimensional data space to two- or three-dimensional data space. **Figure 1A** illustrates the performances of principal component analysis (PCA) and tSNE on multiple datasets. It is clear that tSNE, a nonlinear method, can usually achieve better visualization effects than PCA, a linear method. This is because tSNE can group the cell points from one class cluster together and keep the cell points from different classes separated from each other. The quantitative measurement of the influence of PCA and tSNE by the Davies-Bouldin index also supported this conclusion, as shown in the supplementary files (**Supp 2**). Of note, due to the large computational complexity of nonlinear methods, the general strategy for large data analysis includes two steps. The first is to reduce the dimension to 20 to 50 by PCA, and the second is to reduce such moderate dimension to 2 to 3 by tSNE. This strategy is expected to achieve a good balance between computational performance and resource consumption.

Second, in the cell clustering analysis, the analyzed genes are selected that exhibit expression in at least three cells, so that most genes have actually been used. For hierarchical clustering, k-means, tSNE+k-means, and SIMLR, the number of clusters for each method was fixed to be the same as the number of biological classes corresponding to each dataset, as shown in **Table 3**. For SNN-Cliq and SEURAT, the parameters were adjusted to guarantee that the number of final clusters was the same as the number of biological classes in those datasets, as shown in **Table 3**. In other words, the number of clusters for every method is the same for one dataset to make different methods fairly comparable to ARI. As seen in **Figure 1B**, it is obvious the performances of tSNE+k-means, SIMLR, and SEURAT were better than those of others with higher ARI values in most scRNA-seq datasets. In addition, although tSNE+k-means, SIMLR, and SEURAT have similar performances with regard to ARI, they usually accurately detected different true classes (**Figure 1C**). This means different methods would have different analysis preferences due to different underlying mathematical or biological frameworks and explanations of scRNA information.

**Figure 2:**
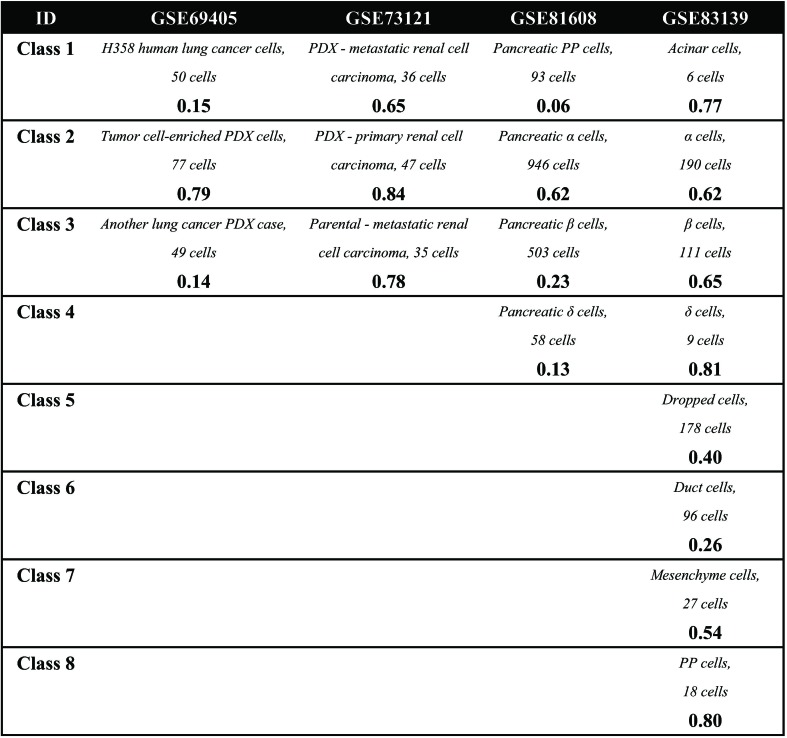
Summary of robustness comparison.

Third, scRNA-seq data follow a time series and the expression of cells may change continuously. For this kind of dataset, some statistical methods can be used to order the cells one by one along a trajectory, which is called pseudo-time or pseudo-trajectory. This mathematical model has been widely applied in developmental biology to reconstruct the differentiation processes and find the key time point of differentiation (Cannoodt et al., 2016). In addition, cell pseudo-time analysis can also be used in studies of cancer and diabetes to reconstruct the occurrence and transformation processes of complex diseases. Thus, the Monocle and DPT have been carried out for pseudo-time analysis on multiple scRNA-seq datasets; these two computational methods are dependent on entirely different principles. In this cell pseudo-time analysis, the most expression-variable genes are selected as feature genes for downstream analysis. As shown in **Figure 1D**, the feature genes exhibit great differences between datasets with different biological backgrounds; however, the two datasets on similar biological phenotypes still have much overlap (i.e., the feature genes from two datasets related to tumor cells with treatments or those from two datasets associated with diabetes). Of note, using human pancreas scRNA-seq datasets in another platform (i.e., GSE86469 and GSE81547; **Table 2**) as controls, the top 50 selected feature genes from the total four datasets indeed had more overlapping genes, as listed in the supplementary files (**Supp 3**). In **Figure 1E**, it is seen that both Monocle and DPT are able to reconstruct the pseudo-time with branches, and DPT seems to obtain more accurate results as the cells of the same cell type tend to group together. Meanwhile, the pseudo-time and branch point seem to be clearer in the analyses of Monocle. Of note, the performance of pseudo-time analysis will be strongly influenced by the selected feature genes. In this comparison, the most expression-variable genes were used, but usually it would be much better to select the feature genes based on the prior biological knowledge in each case study. Furthermore, the consistency of pseudo-time results from different methods is considered and evaluated. As shown in **Figure 2**, the correlations between the first principal components of the pseudo-time results from Monocle and DPT have been calculated. Then, the estimation similarities of cell orders in particular cell classes from different methods are compared. It is obvious that the cell order correlations have huge variances in a wide range among different prior-known cell classes. In addition, two other pseudo-time methods, Wanderlust (Bendall et al., 2014) and SCUBA (Marco et al., 2014), were also applied to reconstruct the pseudo-time trajectory of single cells without branch, as discussed in the supplementary files (**Supp 4**). The observations and conclusions were similar. Thus, in the pseudo-time analysis, consensus performance of dissimilar methods is weak currently.

## Conclusion

scRNA-seq has opened a new way to study complex biological phenomena on the single-cell level, which will be especially helpful in the research of complex diseases. However, to enhance its performance in actual applications, e.g., in the clinic, several improvements are still required. For cell clustering and identification, gene networks rather than separate genes would be more important and reliable to characterize cell states (e.g., network biomarkers for disease subtypes) (Zeng et al., 2014; Zeng et al., 2016). For the cell order, the start or end point of pseudo-time is still a manual judgment, and the auto-determination of these time points will render these methods more flexible and applicable (e.g., temporal driving for disease causality) (Yu et al., 2017; Wang et al., 2018; Setty et al., 2019). The branch point of pseudo-time also requires more models on critical transitions (e.g., tipping point for disease transition) (Zeng et al., 2013; Li et al., 2014). Particularly, the assembling method with good consensus on different datasets is expected to provide more robust integrative scRNA-seq methods for biological and biomedical studies (e.g., pattern fusion for disease heterogeneity) (Shi et al., 2017; Guo et al., 2018).

## Author Contributions

TZ conceived the concept and design of the work. HD and TZ performed the experiments. TZ and HD analyzed the results. TZ drafted the manuscript. TZ and HD revised the paper.

## Funding

This study was supported by the National Key R&D Program Special Project on Precision Medicine (2016YFC0903400), the National Natural Science Foundation of China (11871456 and 61803360), the Shanghai Municipal Science and Technology Major Project (2017SHZDZX01), and the Natural Science Foundation of Shanghai (17ZR1446100).

## Conflict of Interest Statement

The authors declare that the research was conducted in the absence of any commercial or financial relationships that could be construed as a potential conflict of interest.

## Supplementary Material

Supp 1 Parameter settings of scRNA-seq analysis methods.

Supp 2 Feature genes of four human pancreas scRNA-seq datasets.

Supp 3 Quantitative measurement of PCA and tSNE.

Supp 4 Additional pseudo-time methods on four scRNA-seq datasets.
