# Supplementary material for: Single-Cell RNA Sequencing-Based Computational Analysis to Describe Disease Heterogeneity
Source: Front Genet. 2019 Jul 12;10:629. doi: 10.3389/fgene.2019.00629 (PMC6640157; doi:10.3389/fgene.2019.00629)
Supplement: Supp 1 — Parameter settings of scRNA-seq analysis methods. [file Table_1.docx]

**Algorithms and their parameters used in clustering analysis**

*Preprocessing*: All datasets are normalized as *log-counts per million*.

*Hierarchical clustering*

Distance between two objects: *Euclidean distance*

Algorithm for computing distance between clusters: *Ward’s linkage* (Inner squared distance, minimum variance algorithm)

Number of clusters: *Same as the number of categories in the original dataset*

*k-means clustering*

Centroid initialization: *k-means++ algorithm*

Distance between two objects: *Squared Euclidean distance*

Maximum number of iterations: *1000*

Number of times to repeat clustering using new initial cluster centroid positions: *1000*

Number of clusters: *Same as the number of categories in the original dataset*

*t-SNE + k-means*

Preprocessed by PCA to reduce the dimensions to: *20*

t-SNE reduce the dimensions to: *5*

Perplexity: *30*

Clustering method: *k-means*

*SIMLR*

Number of clusters: *Same as the number of categories in the original dataset*

Other parameters: *Default*

*SNN-Cliq*

Number of neighbors: *3*

Other parameters: *Default*

*SEURAT*

Preprocessed by PCA to reduce the dimensions to: *10*

Resolution: *Make the number of clusters is the same as the number of categories in the original dataset*

*Monocle2*

All parameters: *Default* (*http://cole-trapnell-lab.github.io/monocle-release/docs*)

*DPT* (*Diffusion Pseudo-time*)

The method used to build the transition matrix: *nearest neighbors* (*nn*)

Number of nearest neighbors for building the nn-graph: *20*

Number of nearest neighbors for adjusting the Gaussian kernel width: *10*

Statistical value for stopping the iterative branch searching: *1.01*
