# Supplementary material for: Single-Cell RNA Sequencing-Based Computational Analysis to Describe Disease Heterogeneity
Source: Front Genet. 2019 Jul 12;10:629. doi: 10.3389/fgene.2019.00629 (PMC6640157; doi:10.3389/fgene.2019.00629)
Supplement: Supp 3 — Quantitative measurement of PCA and tSNE. [file Table_3.docx]

Quantitative measurement of PCA and t-SNE

|  | GSE69405 | GSE73121 | GSE81608 | GSE83139 |
| --- | --- | --- | --- | --- |
| PCA | 0.28 | 1.67 | 4.75 | 6.79 |
| t-SNE | 0.20 | 0.90 | 1.44 | 4.11 |

Here we used Davies-Bouldin index (DBI) to measure the performance of PCA and t-SNE, which is calculated as

$$DBI=\frac{1}{M}\sum_{i=1}^{M} \max_{j\neq i} \left(\frac{\sqrt{\frac{1}{\left|\Omega_{i}\right|}\sum_{X_{k}\in\Omega_{i}} \left\|X_{k}-A_{i}\right\|^{2}}+\sqrt{\frac{1}{\left|\Omega_{j}\right|}\sum_{X_{k}\in\Omega_{j}} \left\|X_{k}-A_{j}\right\|^{2}}}{\left\|A_{i}-A_{j}\right\|}\right)$$

where the *N* points (or cells) *X*_1_, *X*_2_ … *X_N_* are classified into *M* groups Ω_1_, Ω_2_ … Ω*_M_*, and the centroid of each group is *A*_1_, *A*_2_ … *A_M_* respectively, |Ω*_i_*| represents the number of points in Ω*_i_*, ||·|| represents Euclidean distance. **As DBI is defined as the ratio of the within cluster scatter to the between cluster separation, lower value of DBI means that the clustering is better.**
