# Supplementary material for: Single-Cell RNA Sequencing-Based Computational Analysis to Describe Disease Heterogeneity
Source: Front Genet. 2019 Jul 12;10:629. doi: 10.3389/fgene.2019.00629 (PMC6640157; doi:10.3389/fgene.2019.00629)
Supplement: Supp 4 — Additional pseudo-time methods on four scRNA-seq datasets. [file Table_4.docx]

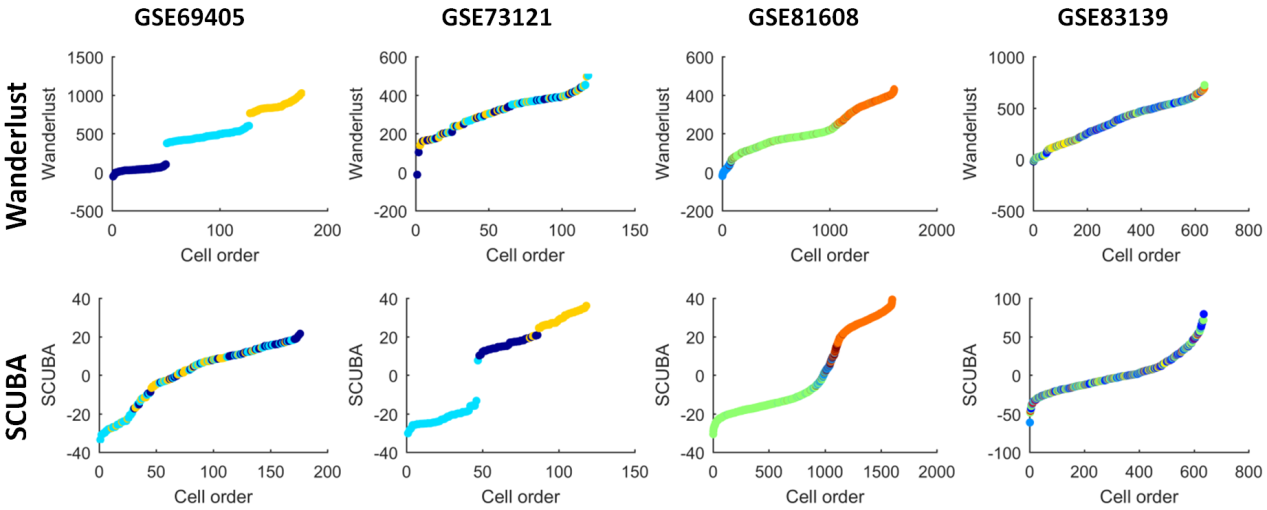


We have carried on two additional pseudo-time methods Wanderlust [1] and SCUBA [2] to reconstruct the pseudo trajectory of single cells without branch respectively. The difference of the two methods is more obvious than the results discussed in main text:

1. For cancer cells, Wanderlust shown clear cell types/groups on the cell order in data GSE69405, meanwhile, SCUBA shown better discrimination of cell types on cell order in data GSE73121;
2. For Diabetes cells, Wanderlust and SCUBA seem to both have better performance on data GSE81608 than those on data GSE83139.

Thus, these additional results supported again, in the pseudo-time analysis, consensus performance of dissimilar methods is low.

**Parameters:**

Wanderlust:

Preprocessed by PCA to reduce the dimensions to: *50*

Other parameters: *Default*

SCUBA:

All parameters: *Default*

**References:**

[1] Bendall, S. C., Davis, K. L., Elad, A., Tadmor, M. D., Simonds, E.F., Chen, T. J., et al. (2014). Single-cell trajectory detection uncovers progression and regulatory coordination in human b cell development. *Cell*, **157(3)**, 714-25.

[2] Marco, E., Karp, R. L., Guo, G., Robson, P., Hart, A. H.,Trippa, L., et al. (2014). Bifurcation analysis of single-cell gene expression data reveals epigenetic landscape. *Proceedings of the National Academy of Sciences of the United States of America*, **111(52)**, 5643-50
